# Effect of a fat spread enriched with medium-chain triacylglycerols and a special fatty acid-micronutrient combination on cardiometabolic risk factors in overweight patients with diabetes

**DOI:** 10.1186/1743-7075-8-21

**Published:** 2011-04-08

**Authors:** Roswitha Siener, Christina Ehrhardt, Norman Bitterlich, Christine Metzner

**Affiliations:** 1 Medical Nutrition Science, Department of Urology, University of Bonn, Bonn, Germany; 2 Bonn Education Association for Dietetics r. A., Cologne, Germany; 3 Medicine and Service Ltd, Department of Biostatistics, Chemnitz, Germany; 4 Department of Internal Medicine III, University Hospital, RWTH, Aachen, Germany

## Abstract

**Background:**

Medium-chain triacylglycerols (MCT), omega-3 polyunsaturated fatty acids (n-3-PUFA) and micronutrients may be useful for weight and cardiometabolic risk management. However, studies analyzing the effect of a combination of both in individuals at increased cardiometabolic risk are lacking. Therefore, this randomized, controlled, double-blind study investigated the effect of a fat spread enriched with two different doses of MCT and a special long-chain fatty acid-micronutrient combination on cardiometabolic risk factors in overweight diabetic patients.

**Methods:**

Fifty-four patients received either a fat spread with 6 g/d MCT (MCT30%) or 1.2 g/d (MCT6%). Forty-three completed the study. Analysis was performed according to the median of MCT intake (supplemented and food-derived MCT). Clinical, anthropometric, blood, 24 h-urine parameters and dietary intake were assessed at baseline and after 12 weeks.

**Results:**

Total MCT intake > 7 g/d (MCT > 7 group) significantly reduced waist circumference (WC) by 1.81 ± 2.69 cm, whereas ≤ 7 g/d MCT (MCT ≤ 7 group) increased WC by 0.32 ± 3.03 cm (p = 0.027), which was supported by a change in waist-to-height ratio (WHtR) (p = 0.018). Fasting serum triglycerides (TG) increased in both groups over time due to dietary habits. In contrast, diabetic metabolic situation and urinary albumin excretion did not alter. Urinary pH differed significantly between groups after 12 weeks.

**Conclusion:**

An intake of >7 g/d MCT reduced WC in overweight diabetics, whereas the increase in the intake of fatty acids may have worsened fasting TG. Therefore, the suitability of a fat for nutrient enrichment remains to be challenged, and further studies in low-fat matrices are desirable.

## Introduction

Evidence indicates the importance of abdominal adipose tissue as endocrine tissue being key for cardiometabolic risk factors [1]. Among patients with type 2 diabetes the combination of abdominal enlargement and hypertriglyceridemia, so-called hypertriglyceridemic waist (HW), is highly prevalent [2] and has been associated with a greater degree of subclinical atherosclerosis that may be related to the proatherogenic lipoprotein changes [3,4]. These lipoprotein changes called atherogenic dyslipidemia are typically characterized by reduced concentrations of HDL cholesterol (HDL-C), elevated triglycerides (TG), and elevated small, dense LDL particles [5]. HW has also been proposed to be an important factor increasing C-reactive protein (CRP) concentrations and relative coronary risk in patients with type 2 diabetes of any age and sex [6,7]. Moreover, reactive oxygen species are produced in various tissues under diabetic conditions leading to an antioxidant depletion and increased lipid oxidation, advanced glycation end products (AGE), cell damage and endothelial dysfunction [8,9].

A supplementation of medium-chain triacylglycerols (MCT) for conventional dietary fats has been proposed as beneficial for weight management, since MCT are rapidly absorbed and preferentially transported through the portal venous system to the liver. The subsequent stimulation of β-oxidation in hepatocytes may reduce the circulating fatty acids available to the adipocytes [10]. Moreover, MCT enhances energy expenditure following thermogenesis [11-13].

The cardiometabolic protective effects of omega-3 polyunsaturated fatty acids (n-3-PUFA) appear to be due to a synergism between multiple mechanisms. These involve anti-inflammatory, inflammation-resolving, anti-arrhythmic and anti-thrombotic effects as well as a regulation of transcription factors, gene expression and membrane fluidity [14]. Moreover, n-3-PUFA, in particular eicosapentaenoic acid (EPA) and docosahexaenoic acid (DHA), have been demonstrated to exert beneficial effects in lowering TG concentrations [15], including in patients with atherogenic dyslipidemia associated diabetes [16,17].

Since the fat spreads were also enriched with vitamins and minerals, their consumption may add to an increase in the daily supply of these essential micronutrients, and thus ameliorate nutritional status. These changes may exert favourable effects on weight management and cardiometabolic risk factors [18,19].

As fat spread is a central source of fat in the diet, the choice of a spread providing a high-quality fat and micronutrient profile may be an important dietary measure for patients with diabetes. Fat spreads characterized by a combination of MCT, n-3-PUFA and micronutrients have not been evaluated so far in this cohort. Therefore, aim of this study was to assess the benefit of a fat spread characterized by the above mentioned combination on cardiometabolic risk factors in overweight diabetic patients.

## Methods

### Participants

Overweight diabetic patients were recruited from the region of Altomuenster, Bavaria, Germany. Both men and women aged 30 to 82 years with a body mass index (BMI) of 27 kg/m^2 ^or greater and a waist circumference (WC) ≥94 cm for women and ≥102 cm for men were included into the study. Overweight diabetic patients were defined by clinical criteria. Individuals taking any medication or nutritional supplements for weight reduction were excluded. Further exclusion criteria comprised the supplementation and/or therapy with marine n-3-PUFA, micronutrients, treatment with glitazones and/or telmisartan, insulin-dependent diabetes, ketoacidosis and acute or chronic diarrhea. The study was approved by the Ethics Committee of the Bavarian Chamber of Physicians, Munich, Germany, and all patients provided informed consent before study onset.

### Study design

This prospective study was carried out in a double-blind controlled manner. Patients were randomly assigned to receive a fat spread with either 6 g/d (MCT30%) or 1.2 g/d MCT (MCT6%), which were equally enriched with special unsaturated fatty acids and micronutrients (Table 1). Patients were asked to consume 2 × 15 g/d of the fat spreads (MCT30% or MCT6%, respectively) for 12 weeks as substitution for their usual dietary spread and to maintain their common diet and physical activity level during the study.

**Table 1:** Mean composition of the MCT30% and MCT6% fat spread per daily serving (2 × 15 g)^1^

	MCT30%	MCT6%
Energy (kcal)	176	176
Carbohydrates (g)	0	0
Protein (g)	0	0
Fat (g)	19.5	19.5
		
Fatty acids		
SAFA (g)	8.7	7.2
- MCT (g)	6.0	1.2
- SAFA without MCT (g)	2.7	6.0
MUFA (g)	6.9	8.1
- Oleic acid (g)	6.6	7.8
PUFA (g)	3.9	4.2
- LA (g)	2.88	3.1
- ALA (g)	0.87	0.9
- EPA (g)	0.15	0.15
- DHA (g)	0.09	0.09
Trans fatty acids (g)	0.12	0.09
Cholesterol (g)	0.006	0.006
		
Micronutrients		
Vitamin A (μg RE)	240	240
Vitamin B1(mg)	0.45	0.45
Vitamin B2 (mg)	0.48	0.48
Nicotinic acid (mg)	4.95	4.95
Vitamin B6 (mg)	0.48	0.48
Folic acid (μg)	120	120
Vitamin B12 (μg)	1.95	1.95
Vitamin D3 (μg)	1.8	1.8
Vitamin E (mg)	3.3	3.3
Sodium (g)	0.002	0.002
Chrome (mg)	0.015	0.015
Manganese (mg)	0.6	0.6

^1 ^abbreviations used: ALA, alpha-linolenic acid; EPA, eicosapentaenoic acid; DHA, docosahexaenoic acid; LA, linolenic acid; MCT, medium-chain triacylglycerols; MUFA, monounsaturated fatty acids; PUFA, polyunsaturated fatty acids; RE, retinol equivalent; SAFA, saturated fatty acids

Data were analyzed at baseline and 12 weeks of intervention. Body weight (kg), height (cm) and WC (cm) were measured. WC was determined to the nearest 0.1 via flexible tape and measurement was documented by photography. Anthropometric measurements and venous blood samples were performed in the morning after an overnight fasting period of at least 12 h. Dietary intake data (3-day food record) was analyzed using PRODI 5.5 software (WVG, Stuttgart, Germany). The average of three days was assessed. Glomerular filtration rate (GFR) was calculated using the Modification of Diet in Renal Disease (MDRD) study equation [20]. Compliance was monitored by weighing of returned spread tubs at week 8 and 12 of intervention as well as by evaluating dietary records on which the number of spread servings per day were recorded. Analysis was performed according to the median of MCT intake (supplemented and food-derived MCT), which was 7 g/d MCT.

### Laboratory methods

SYSCOMP GmbH, Augsburg, Germany, conducted all laboratory analyses. Analysis of serum glucose (hexokinase method), uric acid (uricase-PAP method), gamma-glutamyltransferase (GGT), aspartate-aminotransferase (ASAT), alanine-aminotransferase (ALAT) (IFCC method), glycated haemoglobin (HbA1c) (turbidimetric immunologic inhibition assay (TINIA)), insulin (ECLIA), total cholesterol (TC) (CHOD-PAP method), LDL-C and HDL-C (enzymatic colour test), TG (GPO-PAP method), CRP sensitive (turbidimetry) and 24 h urinary albumin (turbidimetry), pH (Combur test) and sodium (ion sensitive electrode (ISE)) were measured on Roche analyser.

### Statistical methods

All data are presented as means ± standard deviation. Significance was set at p-value 0.05 (two-sided). Changes of WC and body weight from baseline were determined using non-parametric analyses (exact Mann-Whitney-U-test). Pre-post intervention changes of all other variables were analyzed via non-parametric Wilcoxon test and inter-group differences were compared using Mann-Whitney-U test. ANCOVA including use of WC change as a covariate was used to control for the potential confounder of serum fasting TG. Data analysis was performed based on the per protocol population and by using SPSS^® ^for Windows (version PASW 18.0).

## Results

### Participants

A total of 54 overweight diabetic patients were recruited, of whom 43 were included into the per protocol (PP) population (23 men and 20 women). Reasons for an exclusion from the PP population included poor compliance as determined by more than 20% variance of product consumption (n = 7) and variation in study duration of more than 7 days (n = 2). Moreover, 2 patients left the study due to personal reasons (additional file 1). Both fat spreads were well tolerated by the patients and the majority of patients followed the treatment without any reported difficulty.

Male and female subjects were equally distributed (12 and 9 in the MCT > 7 group, 11 and 11 in the MCT ≤ 7 group, respectively; p = 0.639). Metformin was used as anti-diabetic medication by 20 and sulfonylurea by 13 patients. Statins were taken as cholesterol-lowering medication by 11 and antihypertensive medication (beta-blocker, angiotensin converting enzyme inhibitors and calcium channel blocker) by 49 patients.

### Clinical measurements

A total MCT intake > 7 g/d (n = 21) significantly reduced WC by 1.81 ± 2.69 cm (p = 0.005), whereas no significant change was observed in the MCT ≤ 7 group (n = 22) (0.32 ± 3.03 cm, p = 1.000) over time (Table 2). The inter-group changes differed significantly (p = 0.027). Additionally, a significant difference in waist-to-height ratio (WHtR) was observed between groups (p = 0.018), whereas BMI and body weight changes did not change significantly. Systolic and diastolic blood pressure remained unaffected by the supplementation in both groups.

**Table 2:** Clinical and biochemical characteristics at baseline and after supplementation^1,2^

	MCT intake > 7 g/d (n = 21)				MCT intake ≤ 7 g/d (n = 22)				**P**^**b**^
	
	wk 0	wk 12	wk 12 - 0	**P**^**a**^	wk 0	wk12	wk 12 - 0	**P**^**a**^	
Age (years)	67.3 ± 9.3				66.3 ± 7.2				0.512
Weight (kg)	95.7 ± 21.2	95.3 ± 21.7	-0.35 ± 1.92	0.641	87.7 ± 11.6	88.3 ± 11.7	0.54 ± 1.76	0.118	0.177
BMI (kg/m^2^)	34.35 ± 6.84	34.23 ± 7.08	-0.12 ± 0.70	0.681	31.69 ± 4.05	31.89 ± 4.10	0.20 ± 0.61	0.092	0.174
WC (cm)	112.5 ± 12.5	110.7 ± 12.9	-1.81 ± 2.69	0.005	106.4 ± 6.6	106.7 ± 7.0	0.32 ± 3.03	1.000	0.027
WHtR	0.676 ± 0.076	0.665 ± 0.079	-0.010 ± 0.016	0.008	0.640 ± 0.045	0.642 ± 0.049	0.002 ± 0.018	0.920	0.018
SBP (mmHg)	138.6 ± 18.4	137.9 ± 12.0	-0.7 ± 13.2	0.792	148.4 ± 18.6	142.0 ± 17.2	-6.4 ± 15.4	0.069	0.249
DBP (mmHg)	84.8 ± 11.7	84.1 ± 7.7	-0.7 ± 10.3	0.707	87.7 ± 8.7	85.2 ± 8.7	-2.5 ± 9.2	0.170	0.612
Fasting TG (mg/dl)	147.3 ± 92.0	181.6 ± 122.7	34.2 ± 72.6	0.021	166.5 ± 113.3	216.6 ± 177.1	50.1 ± 108.1	0.022	0.990
TC (mg/dl)	209.5 ± 41.0	214.0 ± 41.6	4.52 ± 18.60	0.177	217.7 ± 37.9	225.4 ± 42.7	7.68 ± 23.04	0.058	0.527
LDL-C (mg/dl)	128.8 ± 34.9	130.7 ± 32.3	1.95 ± 16.59	0.357	136.4 ± 34.9	135.6 ± 40.1	-0.86 ± 22.04	0.848	0.618
HDL-C (mg/dl)	52.1 ± 10.8	49.7 ± 10.6	-2.39 ± 8.75	0.230	49.5 ± 9.9	51.4 ± 13.9	1.88 ± 6.71	0.363	0.085
TC/HDL-C	4.15 ± 0.98	4.50 ± 1.37	0.35 ± 0.82	0.064	4.51 ± 1.01	4.65 ± 1.50	0.14 ± 0.70	0.637	0.289
Non-HDL-C (mg/dl)	157.4 ± 40.3	164.3 ± 43.5	6.91 ± 18.8	0.058	168.2 ± 35.6	174.0 ± 42.8	5.80 ± 22.7	0.158	0.981
Fasting glucose (mg/dl)	127.5 ± 21.8	132.0 ± 29.7	4.48 ± 19.91	0.422	124.4 ± 23.9	126.5 ± 34.6	2.09 ± 18.70	0.676	0.313
Fasting insulin (μU/ml)	12.6 ± 9.1	13.7 ± 11.0	1.09 ± 9.33	0.509	14.4 ± 6.8	17.6 ± 25.4	3.17 ± 24.16	0.299	0.177
HOMA-Index	4.14 ± 3.73	4.81 ± 5.52	0.68 ± 5.21	0.639	4.46 ± 2.39	5.72 ± 9.01	1.26 ± 8.68	0.306	0.234
HbA1c (%)	6.76 ± 0.58	6.69 ± 0.78	-0.067 ± 0.380	0.367	6.46 ± 0.47	6.46 ± 0.53	0.004 ± 0.208	0.844	0.179
CRP (mg/l)	6.17 ± 5.97	5.52 ± 4.91	-0.66 ± 2.77	0.140	5.80 ± 8.46	4.45 ± 4.63	-1.35 ± 6.76	0.274	0.961
Uric acid (mg/l)	5.96 ± 1.53	6.27 ± 1.52	0.31 ± 0.66	0.024	6.58 ± 1.26	6.58 ± 1.04	0.00 ± 1.09	0.944	0.103
GGT (U/l)	43.8 ± 50.1	41.2 ± 26.4	-2.57 ± 27.84	0.265	43.8 ± 47.6	44.2 ± 30.5	0.45 ± 20.82	0.023	0.442
ASAT (U/l)	28.4 ± 10.6	28.0 ± 8.3	-0.43 ± 5.97	0.861	28.1 ± 11.9	27.1 ± 6.5	-1.00 ± 9.01	0.480	0.643
ALAT (U/l)	33.5 ± 21.8	31.2 ± 14.3	-2.24 ± 9.96	0.650	29.2 ± 14.1	30.4 ± 13.8	1.18 ± 10.77	0.166	0.607
GFR (mL/min/1.73 m^2^)	87.9 ± 19.1	86.4 ± 22.4	-1.54 ± 11.28	0.433	81.7 ± 15.7	83.4 ± 14.9	1.71 ± 8.60	0.095	0.290
U-Albumin (mg/24 h)	55.3 ± 199.4	19.5 ± 39.9	-35.8 ± 162.7	0.594	62.1 ± 225.8	60.0 ± 150.1	-2.1 ± 109.3	0.641	0.842
U-pH (24 h)	5.81 ± 0.81	5.52 ± 0.73	-0.29 ± 0.70	0.062	5.80 ± 1.08	6.05 ± 1.12	0.25 ± 0.88	0.176	0.032
U-Sodium (mmol/24 h)	212.0 ± 84.6	234.5 ± 89.0	22.5 ± 78.2	0.145	208.6 ± 67.6	231.0 ± 75.6	22.4 ± 72.2	0.153	1.000

^1 ^data are presented as mean ± SD

^a ^p values for comparison between week 0 and 12 (Wilcoxon-Test)

^b ^p value for comparison of group × time interactions (Mann-Whitney-U-Test)

^2 ^abbreviations used: ASAT, aspartate-aminotransferase; ALAT, alanine-aminotransferase; CRP, C reactive protein; DBP, diastolic blood pressure; GFR, glomerular filtration rate; GGT, gamma-glutamyltransferase; HbA1c, glycated haemoglobin; HDL-C, HDL cholesterol; LDL-C, LDL cholesterol; MCT, medium-chain triacylglycerols; SBP, systolic blood pressure; TC, total cholesterol; TG, triglycerides; WC, waist circumference; WHtR, waist to height ratio; U-albumin, urinary albumin; U-pH, urinary pH; U-sodium, urinary sodium

### Serum parameters

The average value of fasting TG was below the cut-off level of 150 mg/dL in the MCT > 7 group (147.3 ± 92.0 mg/dL), whereas the fasting triglyceride level of the MCT ≤ 7 group was hypertriglyceridemic (166.5 ± 113.3 mg/dL) (Table 2). Over time, fasting TG increased significantly in both groups (p = 0.021 and p = 0.022, respectively), but there was no inter-group difference. Total cholesterol (TC), LDL-C, HDL-C, non-HDL-C and TC/HDL-C ratio did not change during supplementation. Fasting glucose, insulin, HOMA index, HbA1c and CRP were elevated in both groups during the study, but were not affected by intervention. The same applies to ASAT and ALAT, however GGT increased significantly in the MCT ≤ 7 group (p = 0.023). In the MCT > 7 group, uric acid increased significantly during supplementation (p = 0.024), but remained within the normal range.

### Urine parameters

Whereas no changes were observed for GFR, urinary albumin and sodium excretion, urinary pH varied significantly between groups after 12 weeks of intervention (p = 0.032) (Table 2).

### Dietary intake

Except for the MCT intake, there was no significant difference in macro- and micronutrient consumption between both groups (Table 3). However, the relation of macronutrients shifted due to changes in dietary habits. Fat intake and percentage of energy consumed from fat increased during supplementation, however this change was not significant in the MCT > 7 group. Saturated fat intake was found to be significantly elevated in the MCT > 7 group by 7.3 ± 11.7 g/d (p = 0.016), mainly due to supplementation. PUFA consumption increased significantly only in the MCT > 7 group (p = 0.033), whereas monounsaturated fatty acid (MUFA) intake, including oleic acid, was significantly higher following fat spread consumption in both groups (p < 0.039 and 0.010, respectively). The same applied for daily alpha-linolenic acid (ALA), EPA and DHA intake. Glucose, fructose, sorbitol and purine intake remained stable over time. However, the fat spread consumption affected daily vitamin supply, as revealed by a significant increase in vitamin D, B2 and folic acid intake after 12 weeks in both groups. Vitamin E intake rose in the MCT > 7 group, and nicotinic acid as well as vitamin B6 consumption were significantly enhanced over time in the MCT ≤ 7 group, without significant differences between groups.

**Table 3:** Daily dietary intake at baseline and after supplementation^1,2^

	MCT intake > 7 g/d (n = 21)				MTC intake ≤7 g/d (n = 22)				**P**^**b**^
	
	wk 0	wk 12	wk 12 - 0	**P**^**a**^	wk 0	wk 12	wk 12 - 0	**P**^**a**^	
Energy (kcal/d)	2341.9 ± 650.0	2440.4 ± 758.4	98.5 ± 601.3	0.566	2234.4 ± 950.3	2518.8 ± 1095.4	284.5 ± 1229.6	0.223	0.593
Protein (g/d)	95.6 ± 22.7	93.4 ± 40.7	-2.2 ± 37.9	0.498	90.6 ± 45.7	102.7 ± 57.6	12.1 ± 67.3	0.263	0.211
Protein (%EN)	16.89 ± 2.23	15.43 ± 2.90	-1.47 ± 2.92	0.027	16.27 ± 3.10	16.13 ± 2.72	-0.14 ± 3.55	0.592	0.198
Carbohydrates (g/d)	237.0 ± 59.3	237.2 ± 62.3	0.2 ± 58.1	0.931	240.9 ± 96.9	241.1 ± 86.0	0.2 ± 109.3	0.808	0.771
Carbohydrates (%EN)	41.42 ± 6.35	40.53 ± 7.14	-0.89 ± 5.83	0.715	44.79 ± 7.74	40.27 ± 7.65	-4.51 ± 7.53	0.014	0.076
Fat (g/d)	93.4 ± 30.4	106.6 ± 37.6	13.2 ± 30.2	0.068	89.1 ± 47.0	111.0 ± 57.6	21.9 ± 60.5	0.050	0.789
Fat (%EN)	36.4 ± 5.5	39.4 ± 4.7	3.03 ± 6.16	0.079	35.1 ± 6.2	39.1 ± 5.8	4.04 ± 6.04	0.006	0.423
Alcohol (g/d)	21.3 ± 35.4	19.3 ± 28.2	-1.96 ± 16.86	0.476	11.8 ± 20.2	17.9 ± 27.6	6.03 ± 24.84	0.227	0.206
Alcohol (%EN)	5.33 ± 7.61	4.66 ± 6.29	-0.67 ± 3.29	0.590	3.86 ± 6.05	4.48 ± 6.58	0.62 ± 5.65	0.872	0.771
Cholesterol (mg/d)	363.2 ± 145.2	393.6 ± 213.7	30.4 ± 160.4	0.543	332.4 ± 189.0	359.7 ± 193.7	27.2 ± 259.2	0.758	0.884
SAFA (g/d)	34.6 ± 13.9	41.9 ± 13.5	7.3 ± 11.7	0.016	32.4 ± 17.7	40.2 ± 18.8	7.8 ± 21.1	0.072	0.942
MCT (g/d)	1.31 ± 0.76	7.74 ± 0.56	6.43 ± 0.83	<0.001	1.08 ± 0.64	4.94 ± 2.12	3.86 ± 2.29	<0.001	<0.001
MUFA (g/d)	34.0 ± 12.3	39.9 ± 16.0	5.9 ± 13.5	0.039	34.1 ± 19.9	45.5 ± 26.5	11.3 ± 27.1	0.010	0.627
PUFA (g/d)	14.5 ± 6.6	16.8 ± 5.7	2.3 ± 6.6	0.033	14.7 ± 7.7	18.2 ± 9.0	3.4 ± 9.5	0.082	0.981
Oleic acid (g/d)	30.1 ± 11.2	35.8 ± 14.4	5.7 ± 12.5	0.039	30.1 ± 17.7	40.7 ± 24.5	10.7 ± 24.6	0.011	0.610
LA (g/d)	12.34 ± 5.76	13.79 ± 4.98	1.45 ± 5.61	0.073	12.91 ± 6.88	15.21 ± 8.09	2.30 ± 8.30	0.322	0.981
ALA (g/d)	1.35 ± 0.82	2.09 ± 0.38	0.74 ± 0.78	0.001	1.15 ± 0.54	2.03 ± 0.66	0.88 ± 0.79	<0.001	0.409
LA/ALA	9.60 ± 1.87	6.47 ± 1.23	-3.14 ± 2.08	<0.001	11.37 ± 3.70	7.22 ± 1.70	-4.15 ± 3.43	<0.001	0.395
EPA (g/d)	0.040 ± 0.038	0.187 ± 0.035	0.147 ± 0.048	<0.001	0.035 ± 0.039	0.178 ± 0.027	0.143 ± 0.049	<0.001	0.981
DHA (g/d)	0.075 ± 0.085	0.192 ± 0.148	0.117 ± 0.167	0.003	0.068 ± 0.116	0.152 ± 0.071	0.083 ± 0.151	0.001	0.752
LA/(ALA+EPA+DHA)	8.72 ± 1.63	5.50 ± 1.11	-3.22 ± 1,65	<0.001	10.56 ± 3.67	6.20 ± 1.57	-4.35 ± 3.35	<0.001	0.296
Glucose (g/d)	11.4 ± 6.4	13.7 ± 6.1	2.3 ± 7.0	0.106	14.7 ± 7.4	13.7 ± 8.9	-1.0 ± 8.8	0.661	0.174
Fructose (g/d)	16.7 ± 10.4	17.7 ± 8.8	1.0 ± 11.6	0.532	20.2 ± 9.3	18.0 ± 11.1	-2.1 ± 12.2	0.527	0.296
Sorbitol (g/d)	1.01 ± 0.83	1.25 ± 1.12	0.24 ± 1.37	0.498	1.01 ± 0.58	1.70 ± 1.46	0.69 ± 1.49	0.051	0.437
Retinol (RE, μg/d)	3093.4 ± 6769.2	1671.6 ± 825.4	-1421.8 ± 6808.4	0.434	2611.9 ± 3613.9	1845.3 ± 792.9	-766.5 ± 3442.7	0.783	0.789
Vitamin D (μg/d)	3.09 ± 3.68	5.54 ± 4.76	2.45 ± 6.08	0.010	2.48 ± 3.58	3.54 ± 2.01	1.06 ± 4.36	0.042	0.627
Vitamin E (mg/d)	11.01 ± 5.25	14.45 ± 4.25	3.44 ± 4.66	0.004	12.10 ± 5.65	15.03 ± 7.19	2.92 ± 7.60	0.077	0.481
Vitamin B2 (mg/d)	1.77 ± 0.98	2.18 ± 0.65	0.41 ± 1.03	0.008	1.59 ± 0.72	2.26 ± 1.04	0.67 ± 1.24	0.006	0.752
Nicotinic acid (mg/d)	22.5 ± 8.8	25.9 ± 9.3	3.5 ± 12.2	0.217	19.5 ± 8.3	29.3 ± 15.0	9.8 ± 15.0	<0.001	0.159
Vitamin B6 (mg/d)	2.11 ± 0.80	2.52 ± 1.31	0.41 ± 1.17	0.122	1.98 ± 0.89	2.75 ± 1.31	0.77 ± 1.44	0.002	0.296
Folic acid (μg/d)	250.4 ± 157.3	354.1 ± 88.9	103.7 ± 119.1	0.001	239.5 ± 82.7	371.5 ± 139.4	131.9 ± 153.3	<0.001	0.716
Vitamin B12 (μg/d)	11.14 ± 15.76	10.54 ± 4.74	-0.61 ± 16.15	0.063	8.65 ± 7.70	10.83 ± 5.19	2.18 ± 9.35	0.050	0.369
Vitamin C (mg/d)	92.8 ± 89.2	79.2 ± 29.6	-13.6 ± 86.0	0.848	88.9 ± 40.6	92.3 ± 48.5	3.4 ± 64.1	0.758	0.771
Sodium (g/d)	3.14 ± 1.23	3.02 ± 1.24	-0.12 ± 1.10	0.715	3.39 ± 1.87	3.50 ± 2.21	0.11 ± 2.54	0.485	0.716
Chloride (g/d)	4.74 ± 1.71	4.62 ± 1.84	-0.12 ± 1.51	0.741	5.03 ± 2.65	5.29 ± 3.38	0.26 ± 3.86	0.592	0.644
Salt (g/d)	7.1 ± 2.7	6.9 ± 2.8	-0.2 ± 2.4	0.715	7.5 ± 4.2	7.8 ± 5.1	0.4 ± 5.9	0.509	0.789
Fiber (g/d)	21.9 ± 11.7	22.2 ± 7.2	0.32 ± 8.96	0.434	24.1 ± 10.5	25.3 ± 13.4	1.16 ± 14.47	0.910	0.808
Purins (mg/d)	203.9 ± 67.0	201.9 ± 98.8	-2.0 ± 111.8	0.821	200.7 ± 98.6	230.1 ± 129.3	27.0 ± 145.5	0.327	0.464
Uric acid (mg/d)	613.4 ± 201.4	605.9 ± 297.7	-7.5 ± 336.1	0.794	586.3 ± 279.8	685.2 ± 352.7	98.9 ± 397.4	0.072	0.224

^1 ^data are presented as mean ± SD

^a ^p values for comparison between week 0 and 12 (Wilcoxon-Test)

^b ^p values for comparison of group × time interactions (Mann-Whitney-U-Test)

^2 ^abbreviations used: ALA, alpha-linolenic acid; DHA, docosahexaenoic acid; EN, energy; EPA, eicosapentaenoic acid; LA, linoleic acid; MCT, medium-chain triacylglycerols; MUFA, monounsaturated fatty acids; PUFA, polyunsaturated fatty acids; RE, retinol equivalent; SAFA, saturated fatty acids

## Discussion

To our knowledge this is the first study investigating the effect of a fat spread enriched with MCT and a special fatty acid-micronutrient combination on cardiometabolic risk factors in overweight diabetic patients. The major finding of the present study was a significant decrease in WC in the MCT > 7 group. The reduction in WC observed in this study is in accordance with previous research evaluating the effect of MCT in overweight subjects [21-23]. The supplementation of 5 g/d MCT in form of an oil for instance resulted in a significant decrease in WC compared to the long-chain triacylglycerols (LCT) control treatment (-5.1 ± 3.1 cm vs. -3.3 ± 1.9 cm, p < 0.05) [21]. Tsuji et al. [23] reported a significant reduction in WC by 5.67 ± 0.05 cm with BMI ≥ 23 kg/m^2^, however only in the area of subcutaneous fat, at a total daily MCT intake of 9.24 g/d.

Since some evidence also pointed at a beneficial effect on satiety [24], it has been proposed that under free living conditions an increase in dietary MCT may result in less energy intake and contributes to weight management. However, the fact that we did not find any decrease in energy intake and no change in body weight suggests that the observed effect on WC may be rather ascribed to an effect on energy expenditure and thermogenesis than to an increase in satiety [13,25,26].

Since long-chain PUFA are proposed to reduce TG, we hypothesized that the intervention may involve an improvement in dyslipidemia. However, in our study the consumption of the fat spreads did not result in any reductions in blood lipids. In contrast, we observed a significant rise in fasting TG in both groups after supplementation. It is suggested that fasting TG may have been affected by changes in dietary habits regarding the quantity and quality of fat intake. Fatty acid intake profile of patients revealed a rise in the intake from MUFA, especially oleic acid, in both groups. Table 4 shows that TG-lowering effect could be attributed only to MCT intake, whereas MUFA increased serum TG.

**Table 4:** Serum fasting triglycerides depending on change of fatty acid intake^1^

ANCOVA	Oleic acid (g/d)	MUFA* (g/d)	PUFA (g/d)	MCT (g/d)	SAFA** (g/d)	Age (years)	**Gender**^**2**^ (1 w/2 m)
PP	B: 0.789	B: 2.046	B: -1.710	B: -17.950	B: 0.324	B: -0.050	B: -72.396
	
(n = 43)	95% CI (-9.84;11.42)	95% CI (-44.8;48.9)	95% CI (-19.0;15.6)	95% CI (-42.1;6.2)	95% CI (-13.5;14.2)	95% CI (-6.2;6.1)	95% CI (-175.5;30.7)
	
p = 0.471	p = 0.881	p = 0.930	p = 0.842	p = 0.141	p = 0.962	p = 0.987	p = 0.163

*without oleic acid

**without MCT

^1 ^abbreviations used: MCT, medium-chain triacylglycerols; MUFA, monounsaturated fatty acids; PUFA, polyunsaturated fatty acids; SAFA, saturated fatty acids

^2 ^gender is coded by 1 (women) and 2 (men)

This rise in fat consumption may have counteracted any beneficial effects of the n-3-PUFA and may have caused the adverse effect on fasting TG instead. It further indicates that the spreadable fat has not fully been consumed as a replacement of other fat in the diet as intended, but rather in addition. In deed, several patients reported that the consumption of 30 g/d of spread during the study was much higher than the amount of spread they usually consume which was reflected in their dietary protocols. However, observations in free-living conditions illustrated that users of plant sterol enriched margarines generally consumed less than the 20 g/d recommended by the manufacturers. The average daily intake of phytosterol-enriched margarine ranged between 9 and 14 g [27], indicating that the serving size chosen in this study may not be consistent with habitual spread intake in Europe. Unpredictable effects such as changes in physical activity and nutritional behaviour over time which are difficult to control for may bias results even in carefully designed studies [28].

An additional effect on fasting TG may have been expected due to n-3-PUFA supplementation with the fat spreads. With combined daily intake of 240 mg DHA + EPA and 870/900 mg ALA, the n-3-PUFA intake may have been too low to produce a triglyceride-lowering effect. The recommended daily intake of n-3-PUFA for the treatment of hypertriglyceridemia is 2 to 4 g [15].

The consumption of the fat spreads did not adversely affect fasting glucose, insulin, HOMA index, HbA1c, ASAT or ALAT. As GGT increased in MCT ≤ 7 group, no general effect of MCT supplementation on GGT could be derived.

The observed reduction in urinary pH in the MCT > 7 group may be referred to the fact that MCT have a ketogenic character, as acetyl-CoA produced during medium-chain fatty acid oxidation is directed towards ketone body production [10]. Additionally, oral administration of MCT leads to urinary elimination of C6, C8 and C10 dicarboxylic acid [29,30].

Except for the MCT intake, no significant differences in macro- and micronutrient consumption were observed between both groups. Therefore, it is unlikely that the observed reduction in WC could be related to other nutritional factors than the MCT intake. It is suggested that the micronutrient intake with the fat spread was too low to achieve further beneficial cardio-metabolic effects.

In summary, a daily intake of at least 7 g MCT beneficially affects visceral fat mass, objectivised as WC, in overweight diabetic patients. As the daily intake of MCT through the normal diet is relatively small, a moderate enrichment of food with MCT may effectively contribute to achieve this required intake. The supply of MCT through enriched fat spreads may be adequate for individuals who are used to consume a high amount of spread on a daily basis. But, it may be reasonable to reassess the suitability of a fat spread as matrix for MCT. As dietary counselling for diabetic patients includes recommendations to reduce and modify the fat intake, alternative food matrices such as oil and milk products may be interesting alternatives in order to ensure a healthy overall diet. Since no significant differences in macro- and micronutrient consumption were observed between both groups, it is suggested that any beneficial effects of the n-3-PUFA and vitamins may have been counteracted by the increased intake of other fatty acids.

## Competing interests

This study was supported by a research grant from basis GmbH, Woerthsee, Germany. The funder was neither involved in the study design, interpretation of data, nor in the content of the final manuscript.

None of the authors has a conflict of interest for this manuscript others than that the study has been funded by basis GmbH, Woerthsee, Germany. Prof. Dr. Dr. Metzner was a consultant for basis GmbH, Woerthsee, Germany.

## Authors' contributions

CM designed research. RS was involved in project conception and overall research plan. CE analyzed dietary intake and conducted monitoring. NB performed statistical analysis. RS, CE and CM wrote paper. RS and CM have primary responsibility for final content. All authors read and approved the final manuscript.

## Supplementary Material

Additional file 1 **Trial profile**^**1**^. The trial profile shows flow of the patients from screening to study completion. ^1 ^abbreviations used: ITT, intention to treat, MCT, medium-chain triacylglycerols; PP, per protocol Click here for file
