# Supplementary figures and images for: Effect of a fat spread enriched with medium-chain triacylglycerols and a special fatty acid-micronutrient combination on cardiometabolic risk factors in overweight patients with diabetes
Source: Nutr Metab (Lond). 2011 Apr 8;8:21. doi: 10.1186/1743-7075-8-21 (PMC3090316; doi:10.1186/1743-7075-8-21)

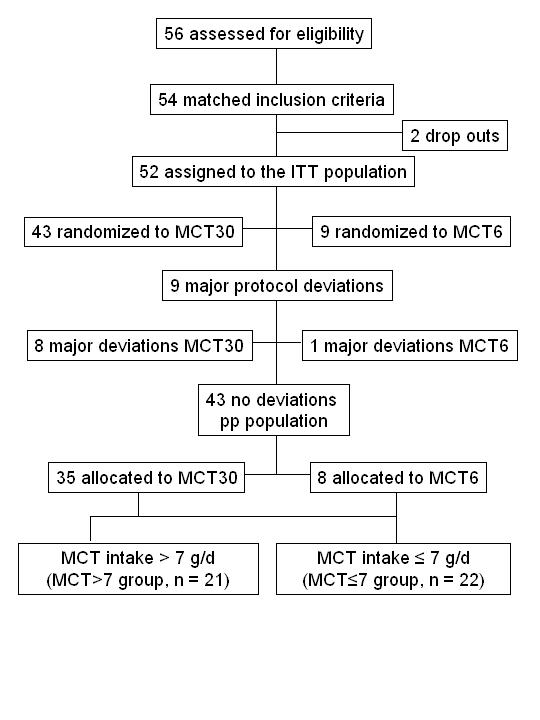

Supplement: Additional file 1 — Trial profile1. The trial profile shows flow of the patients from screening to study completion. 1 abbreviations used: ITT, intention to treat, MCT, medium-chain triacylglycerols; PP, per protocol [file 1743-7075-8-21-S1.JPEG]
